# Bacterial amyloid curli activates the host unfolded protein response via IRE1α in the presence of HLA-B27

**DOI:** 10.1080/19490976.2024.2392877

**Published:** 2024-08-27

**Authors:** Kaitlyn Grando, Shingo Bessho, Kayla Harrell, Kathrine Kyrylchuk, Alejandro M. Pantoja, Sophia Olubajo, Francisco J. Albicoro, Andres Klein-Szanto, Çagla Tükel

**Affiliations:** a Center for Microbiology and Immunology, Lewis Katz School of Medicine, Temple University, Philadelphia, PA, USA; b Histopathology Facility, Fox Chase Cancer Center, Philadelphia, PA, USA

**Keywords:** *Salmonella*, HLA-B27, reactive arthritis, unfolded protein response, curli, autoimmunity

## Abstract

*Salmonella enterica* serovar Typhimurium (STm) causes gastroenteritis and can progress to reactive arthritis (ReA). STm forms biofilms in the gut that secrete the amyloid curli, which we previously demonstrated can trigger autoimmunity in mice. HLA-B27 is a genetic risk factor for ReA; activation of the unfolded protein response (UPR) due to HLA-B27 misfolding is thought to play a critical role in ReA pathogenesis. To determine whether curli exacerbates HLA-B27-induced UPR, bone marrow-derived macrophages (BMDMs) isolated from HLA-B27 transgenic (tg) mice were used. BMDMs treated with purified curli exhibited elevated UPR compared to C57BL/6, and curli-induced IL-6 was reduced by pre-treating macrophages with inhibitors of the IRE1α branch of the UPR. In BMDMs, intracellular curli colocalized with GRP78, a regulator of the UPR. *In vivo*, acute infection with wild-type STm increased UPR markers in the ceca of HLA-B27tg mice compared to C57BL/6. STm biofilms that contain curli were visible in the lumen of cecal tissue sections. Furthermore, curli was associated with macrophages in the lamina propria, colocalizing with GRP78. Together, these results suggest that UPR plays a role in the curli-induced inflammatory response, especially in the presence of HLA-B27, a possible mechanistic link between STm infection and genetic susceptibility to ReA.

## GRAPHICAL ABSTRACT

**Figure:**
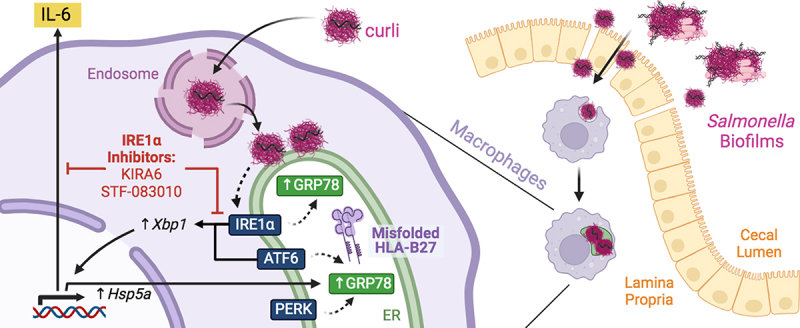

## Introduction

Non-typhoidal *Salmonella enterica* serovar Typhimurium (STm) is a common cause of gastroenteritis worldwide. About 2–4 weeks after STm infection, approximately 4% of patients develop an autoimmune disease called reactive arthritis (ReA),^1^ characterized by joint pain, eye inflammation, and urinary problems. ReA falls under the spondyloarthritis umbrella alongside other autoimmune diseases like ankylosing spondylitis, psoriatic arthritis, and inflammatory bowel disease (IBD), all of which are associated with the HLA-B27 genotype, present in up to 90% of patients.^2–6^

In addition to STm, other invasive enteric bacteria, such as *Shigella*, *Campylobacter*, or *Yersinia*, have been implicated in ReA.^7^ It is thought that ReA pathogenesis may involve biofilms formed by these enterics,^8^ aggregates of bacteria secreting an extracellular matrix for protection and survival during stress. The matrix of STm is composed primarily of the functional amyloid curli, extracellular DNA (eDNA), and polysaccharide cellulose.^9–11^ Curli is the most studied of bacterial amyloids, and is produced by STm, *Escherichia coli*, and other enterics;^12–14^ it is also estimated that up to 40% of biofilms contain similar amyloid proteins.^15^ While *in vitro* conditions for STm biofilm production do not match physiological conditions, nonetheless, we have shown that STm produces curli-containing biofilms in the intestines of infected mice.^16^

Curli is a potent immunogen; curli fibers complex with eDNA in the matrix and activate multiple receptors within phagocytes during uptake, including the toll-like receptor (TLR) 2/1 on the cell surface, the endosomal DNA sensor TLR9, and the cytosolic NLRP3 inflammasome.^17–21^ Curli/DNA provokes the secretion of pro-inflammatory cytokines including IL-6, TNFα, IL-1β, type I IFNs, and IL-17, all of which are associated with systemic inflammation and autoimmunity.^21–23^ Mice intraperitoneally injected with purified curli or infected with wild-type, but not curli-deficient, STm, consistently develop anti-double-stranded DNA autoantibodies and mild knee inflammation,^8,16,23^ implicating a role for curli in the pathogenesis of ReA and other autoimmune diseases.

HLA-B27, an allele of human MHC class I molecules, is present in up to 8% of European and North American people, and rarer in other populations.^24^ HLA-B27 was identified as an important genetic risk factor for ReA as up to 90% of patients with ReA carry this allele.^2–6^ One hypothesis on HLA-B27 pathology is the misfolding of HLA-B27 into nonfunctioning dimers, which may build up in the endoplasmic reticulum and provoke the unfolded protein response (UPR).^2,24,25^ Consistent with this idea, one study found HLA-B27 dimers in HLA-B27+ patients with arthritis, and determined that the presence of HLA-B27 dimers transfected into HeLa cells correlated with enhanced UPR activation.^26^ The UPR functions both to return the cell to equilibrium, by pausing translation and degrading misfolded proteins, but also to invoke a pro-inflammatory response implicated in disease. Briefly, the UPR initiates when glucose regulatory protein 78 (GRP78), also known as BiP and encoded by the *Hsp5a* gene, preferentially binds misfolded proteins, thus dislodging from 3 sensors: PERK, ATF6, and IRE1α, all of which have downstream ties to inflammation. The PERK pathway activates CHOP; produces IL-23, which activates Th17 cells associated with ReA and other spondyloarthropathies;^27^ and activates apoptotic mechanisms.^28–32^ ATF6 increases expression of XBP1, a major downstream transcription factor associated with UPR-induced inflammation.^33^ The IRE1α pathway has the most explored role in inflammation, involving: JNK, NOD1/2, and NFκB activation; indirect activation of the inflammasome; and splicing of XBP1 into its active form which induces further *Hsp5a* expression and cytokines including IL-23, IL-6, TNFα, and IFNs.^28, 34–40^ IRE1α activation can also be enhanced by TLR stimulation.^41,42^ However, given the ubiquity of the HLA-B27 molecule, it is thought that misfolding of the HLA-B27 activates UPR at a low basal level globally, which alone does not provoke a significant inflammatory response, but primes the cells for sensitized reactions to other stimuli.^28^

UPR activation has been described during infection and implicated in the activation of NOD-induced inflammation. While this response is critical for the clearance of some intracellular pathogens like *Chlamydia* and *Citrobacter*, other pathogens like STm utilize ER stress to enhance intracellular growth.^43–47^ HLA-B27 has also been shown to impair intracellular clearance of other *Salmonella* serotypes.^48,49^ In a cohort of patients challenged with *Salmonella* Typhi or Paratyphi A, HLA-B27 genotype was significantly overrepresented in the group susceptible to enteric fever. It was concluded that HLA-B27 misfolding creates an intracellular environment conducive to *S.* Typhi replication, increasing susceptibility to enteric fever.^47^ Furthermore, it was shown that STm utilizes the lipid genesis that occurs as a result of UPR activation to aid in the development of the *Salmonella-*containing vacuole for intracellular survival of the bacteria.^50^

Here, we set out to determine whether curli interacts with the UPR, especially in the presence of HLA-B27 priming, to increase inflammation and autoimmunity.

## Results

### Increased unfolded protein response in macrophages from HLA-B27tg mice *in* *vitro*

**Figure 1.**
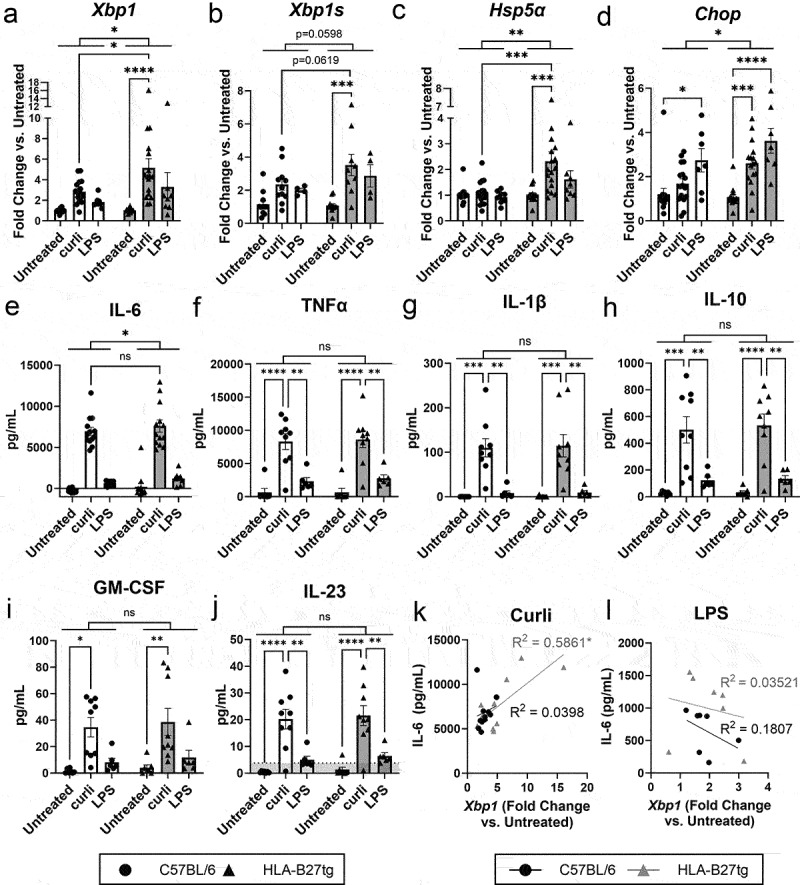
Unfolded protein response and pro-inflammatory cytokines in macrophages from C57BL/6 vs. HLA-B27tg mice.

To determine whether HLA-B27 exacerbates inflammatory responses to curli, we first examined bone marrow-derived macrophages (BMDMs) from HLA-B27tg mice compared to C57BL/6 wild-type controls. While HLA-B27 is present in any nucleated cell,^2,51^ it is especially relevant to examine immune cells like macrophages that could further amplify the inflammatory response. BMDMs from either HLA-B27tg or C57BL/6 mice, were treated with curli or LPS, a potent and well-characterized immunogen, and compared to untreated controls. Curli was purified from the biofilms of STm *msbB* mutant, that produces a modified LPS that does not signal TLR4; additionally, the purification protocol contains washes that strip the remaining LPS.^52^ BMDMs were treated for 24 h, a timepoint which previous studies reported for peak UPR activation.^43–46^ RNA was extracted and analyzed by qPCR for UPR activation, using primers for *Xbp1, Xbp1s* (spliced activated form of *Xbp1*), *Hsp5a*, and *Chop*. Notably, the expression levels of total *Xbp1*, *Xbp1s*, *Hsp5a*, and *Chop* were significantly higher in curli-treated macrophages from HLA-B27tg mice compared to untreated controls (Figure 1(a-d)). Specifically, both *Xbp1* and *Hsp5a* were significantly elevated in curli-treated HLA-B27tg BMDMs compared to curli-treated C57BL/6 BMDMs (Figure 1(a,c)), while the increase in *Xbp1s* in curli-treated groups was just below the threshold for significance (Figure 1(b)). Total *Xbp1* and *Hsp5a* expression showed significant elevation in response to increasing curli concentrations in HLA-B27tg BMDMs compared to C57BL/6 (Supplementary Figure S1(a,c)). Activated spliced *Xbp1s* expression increased dramatically with increasing doses of curli in HLA-B27tg BMDMs but stayed largely steady in C57BL/6 cells, though this change was not statistically significant (Supplementary Figure S1(b)). However, *Chop*, which indicates the PERK branch of UPR, was more responsive to LPS treatment in both HLA-B27tg and C57BL/6 cells. Only HLA-B27tg cells treated with curli exhibited significant low-level activation of *Chop* (Figure 1(d)). ANOVA analysis also indicated a significant increase in the overall activation of *Xbp1*, *Hsp5a*, and *Chop* in the HLA-B27tg group compared to C57BL/6 (Figure 1(a,c-d)). Altogether, these results indicate that curli increases UPR responses especially in the presence of HLA-B27.

BMDMs were cultured from C57BL/6 (black, circles) or HLA-B27tg (gray, triangles) mice. (a-d) qRT-PCR on RNA extracted from BMDMs looking at UPR markers (a) total *Xbp1*, (b) spliced activated *Xbp1s*, (c) *Hsp5a*, (d) *Chop* in cells treated for 24 h with 2.5 μg/mL curli or 100 ng/mL LPS normalized to *Gapdh* and fold change calculated vs. untreated controls. Pro-inflammatory cytokines in BMDM supernatant assessed by (e) IL-6 ELISA or (f-j) MSD U-plex multiplex ELISA including (f) TNFα, (g) IL-1β, (h) IL-10, (i) GM-CSF, and (j) IL-23 (dotted line indicates lower detection limit of the MSD assay). Correlation of secreted IL-6 concentration with *Xbp1* of (k) curli-treated or (l) LPS-treated BMDMs, normalized to untreated controls. BMDMs isolated from *N* = 17 HLA-B27tg mice and 17 C57BL/6 mice cultured in parallel (*N* = 9 HLA-B27tg and 9 C57BL/6 for MSD analysis). [(a-j) Two-way ANOVA with multiple comparisons, (k-l) simple linear regression and probability of non-zero slope (F-test), *= p < 0.05, **= p < 0.01, ***= p < 0.001, ****= p < 0.0001].

### Elevated Xbp1 correlates with elevated IL-6 secretion

Using MSD U-plex, a multiplex ELISA assay, we measured secreted cytokines in the BMDM supernatant. IL-6 was produced at a considerably higher concentration and so was re-analyzed separately using a conventional ELISA assay. Curli treatment produced a strong and significantly elevated cytokine response in both HLA-B27tg and C57BL/6 macrophages, including IL6, TNFα, IL-1β, IL-10, GM-CSF, and IL-23 (Figure 1(e-j)). However, these cytokine responses were not different between curli-treated C57BL/6 and HLA-B27tg macrophages. While ANOVA analysis revealed a significantly higher response overall in HLA-B27tg cells compared to C57BL/6, no difference was noted between the curli-treated samples (Figure 1(e)). Similarly, there was no differential cytokine response between HLA-B27tg and C57BL/6 cells treated with LPS (Figure 1(e-j)).

When *Xbp1* expression, the most prominently altered UPR marker, was plotted against cytokine responses in cells treated with curli, there was a significant positive correlation with IL-6 secretion in HLA-B27tg macrophages, while there was no correlation in C57BL/6 (Figure 1(k)). LPS treatment did not show any correlation between *Xbp1* expression and IL-6 production in either HLA-B27tg or C57BL/6 BMDMs (Figure 1(l)); this is expected as LPS did not significantly activate *Xbp1*. Together, these results suggest that curli triggers UPR activation, particularly in HLA-B27tg cells, but that this increased UPR is not enough to dramatically alter curli-induced cytokine production, except perhaps IL-6 production.

### Inhibiting UPR also decreased pro-inflammatory response to curli

**Figure 2.**
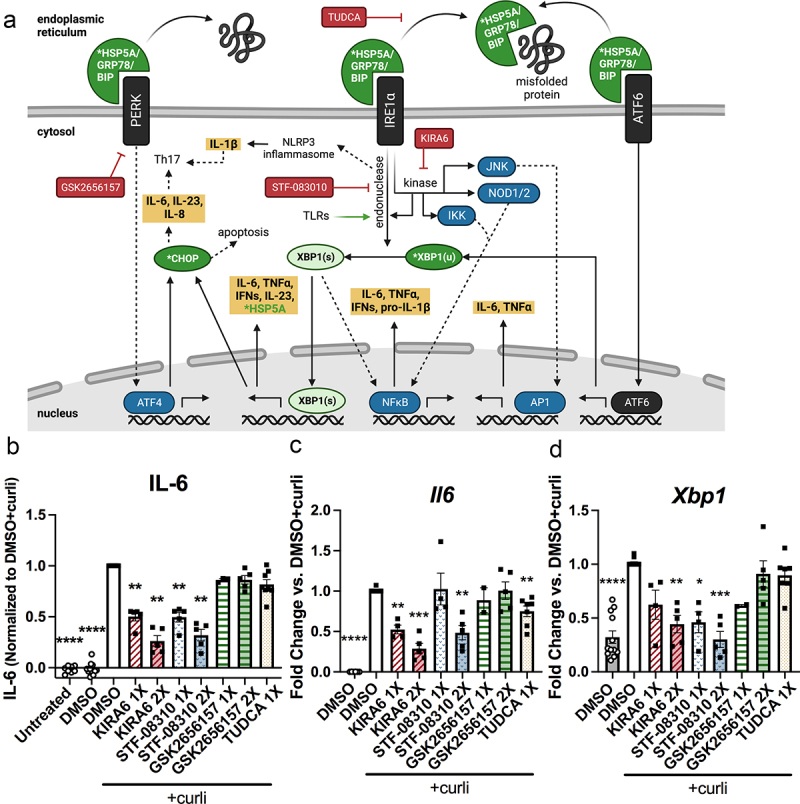
Inhibiting IRE1α of UPR reduces IL-6 response to curli.

To further probe the curli-induced UPR, we utilized the following UPR inhibitors prior to curli treatment of BMDMs: KIRA6 which blocks the kinase activity of IRE1α, STF-083010 which blocks the endonuclease activity of IRE1α, GSK2656157 which inhibits PERK, or TUDCA which inhibits GRP78 dissociation and overall ER stress^38^ (Figure 2(a)). HLA-B27tg BMDMs were pre-treated for 30 minutes with UPR inhibitors then curli was added to determine the effect on the cytokine response. Interestingly, only KIRA6 and STF-083010, which both inhibit the activity of IRE1α, significantly decreased curli-induced IL-6 secretion (Figure 2(b)) and mRNA expression (Figure 2(c)). This result is consistent with both KIRA6 and STF-08310 decreasing *Xbp1* mRNA (Figure 2(d)). TUDCA treatment significantly decreased *Il6* mRNA expression (Figure 2(c)), though to a lesser degree than the IRE1α inhibitors, and this did not correspond to a detectable difference in IL-6 production (Figure 2(b)). GSK2656157, the PERK inhibitor, had no effect on curli-induced IL-6 response (Figure 2(b,c)). These results suggest that the curli-induced IL-6 response involves the UPR in HLA-B27tg BMDMs and is associated with the IRE1α pathway.

(a) Diagram of UPR markers and inhibitors created with Biorender.com. When misfolded proteins are abundant, GRP78, encoded by *Hsp5a*, dislodges from three sensors which then initiate UPR cascades, some of which activate inflammatory pathways and result in pro-inflammatory cytokine secretion. Inflammatory cascades activated by UPR are highlighted here (green = UPR markers, red = inhibitors, black = three UPR-initiating sensors, blue = downstream inflammatory pathways and transcription factors, yellow = downstream cytokine secretion). (b-d) BMDMs from HLA-B27tg mice were pre-treated for 30 minutes with UPR inhibitors: KIRA6 (1X = 1 μM, 2X = 2 μM), STF-08310 (1X = 50 μM, 2X = 100 μM), GSK2656157 (1X = 500 nM, 2X = 1 μM), TUDCA (1X = 200 μM), or DMSO (vehicle control), then treated with 2.5 μg/mL curli or untreated. (b) IL-6 ELISA on BMDM supernatant. (c-d) qRT-PCR on BMDMs for (c) *Il6* or (d) *Xbp1* mRNA expression. *N* = 3-8 HLA-B27tg mice for each inhibitor-treated group, 13 mice total for all trials (not all experiments had 1X and 2X treatments simultaneously). Data normalized to cells treated with DMSO+curli from the same mouse within each replicate experiment and statistically significant deviation from a normalized value of 1 was determined. [(b-d) One sample t-test for significant difference from 1 for data normalized to DMSO+curli group from the same batch of BMDMs, * = p < 0.05, **= p < 0.01, ***= p < 0.001, ****= p < 0.0001)].

### Intracellular curli co-localizes with GRP78

Curli and LPS elicited different responses in HLA-B27tg BMDMs, indicating that the indirect activation of the UPR via TLR stimulation may not be the primary mechanism through which curli induces a UPR-associated pro-inflammatory response. Both LPS and curli are potent stimulators of TLR4 or TLR2, respectively;^18^ while the DNA that irreversibly complexes with curli during biofilm formation activates TLR9.^23^ Studies in human Alzheimer’s disease and Parkinson’s disease suggest direct interaction between amyloid-β or α-synuclein aggregates and the UPR even when added exogenously and outside of the ER itself.^53–56^ Given that curli is structurally similar to human amyloids^57^ and can escape the endosome and localize in the cytosol of macrophages after uptake,^17,23^ we asked if it could directly interact with the UPR.

**Figure 3.**
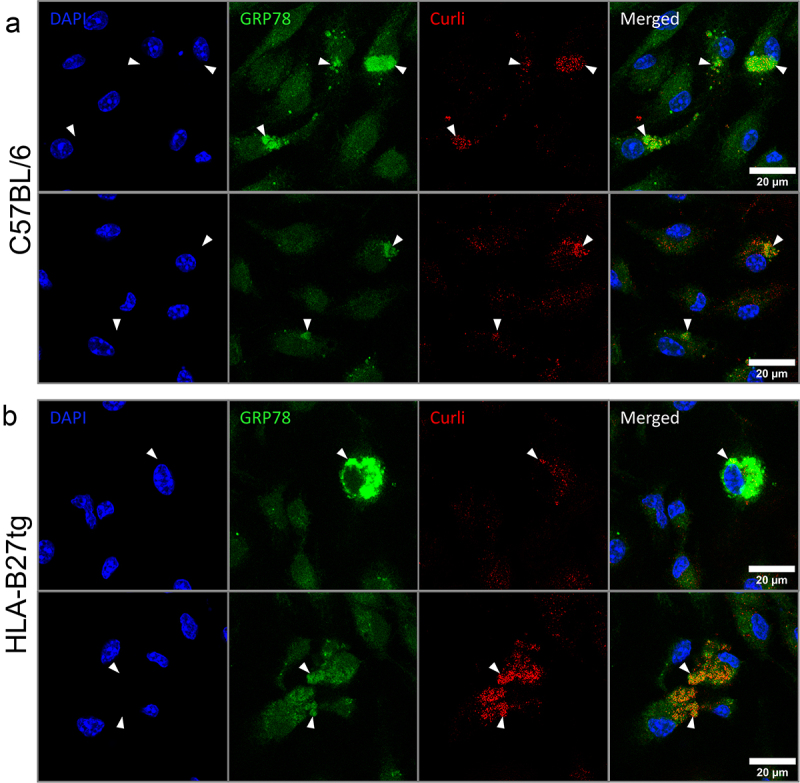
Colocalization of intracellular curli with GRP78.

**Figure 4.**
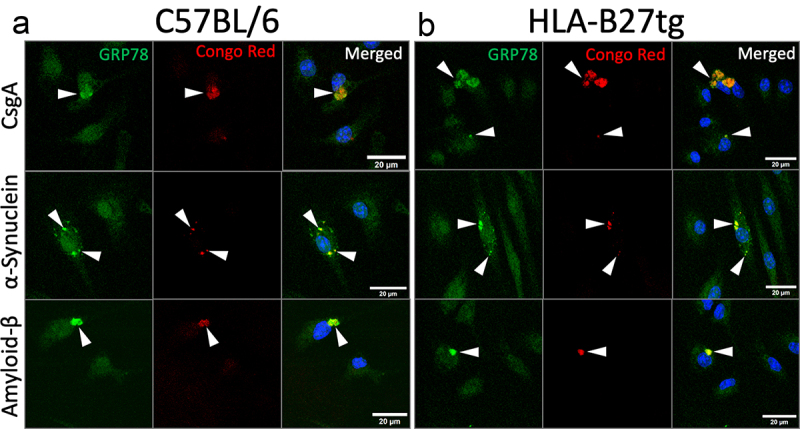
Similar colocalization pattern of other amyloid proteins and GRP78.

To begin addressing this question, we examined BMDMs for colocalization of curli and GRP78, the chaperone protein that preferentially binds misfolded proteins and initiates the UPR inside the ER, encoded by *Hsp5a*.^28^ C57BL/6 and HLA-B27tg BMDMs were treated with Congo red-stained curli for 1 h.^23^ Then cells were fixed and permeabilized, stained for GRP78, and imaged on a confocal microscope. Larger intracellular aggregates of curli consistently colocalized with bright GRP78 staining, in both C57BL/6 and HLA-B27tg BMDMs (Figure 3(a,b)). All cells had baseline staining for GRP78 primarily in the cytoplasm, indicative of all cells having a basal level, likely in the ER. Meanwhile, cells with intracellular curli aggregates also displayed locally elevated GRP78 fluorescence in the same loci as curli. As host amyloids and curli share common characteristics and activate similar pathways, we tested the capacity of α-synuclein and amyloid-β to activate UPR in our assay. We also compared it to synthetic CsgA fibrils, devoid of LPS and DNA. All amyloids tested were stained with Congo-Red prior to treatment. All three amyloids, colocalized with locally elevated GRP78 in both C57BL/6 and HLA-B27tg BMDMs (Figure 4(a,b)). This colocalization and associated elevation of GRP78 in the cytoplasm, whether at the ER membrane or elsewhere, suggests that curli, like host amyloids, may directly interact with the UPR or possibly GRP78 itself.

Representative images of BMDMs grown on coverslips from (a) C57BL/6 or (b) HLA-B27tg mice, treated with 20 μg/mL curli, pre-stained with Congo Red amyloid dye (red), for 1 h, before fixation and staining with anti-GRP78-FITC (green) and nuclei stained with DAPI (blue). White arrows point to colocalization of GRP78 and curli. Scale bars on merged images represent 20 μm, 63X magnification. BMDMs isolated from *N* = 7 C57BL/6 and 8 HLA-B27tg mice.

Representative confocal images of BMDMs cultured from (a) C57BL/6 or (b) HLA-B27tg mice, treated for 1 h with 10 µg/mL recombinant csgA R4-5 (top), α-synuclein (middle), or amyloid-β (bottom) after monomers of each were fibrillized for 48 h and dyed with Congo Red (red). Nuclei were dyed with DAPI (blue) and GRP78 with anti-GRP78-FITC (green). *N* = 4 C57BL/6 and 4 HLA-B27tg mice. Scale bars represent 20 μm, 63X magnification.

### Elevated GRP78 staining in HLA-B27tg BMDMs

**Figure 5.**
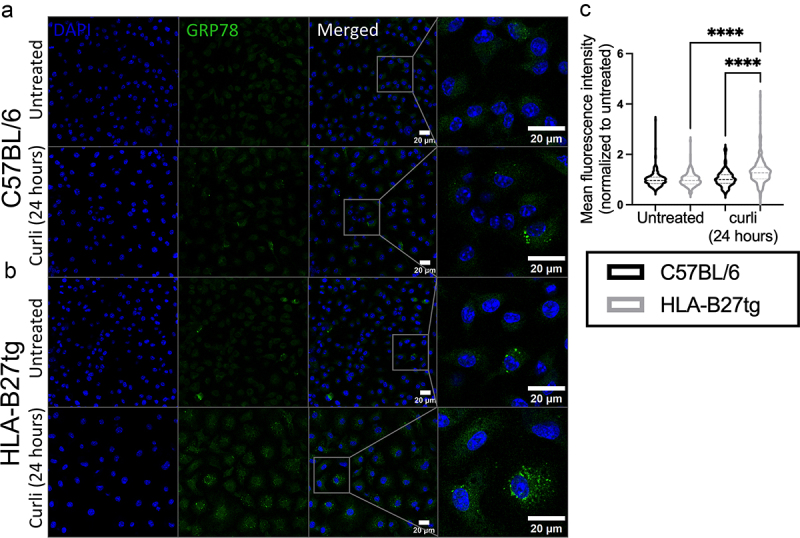
Increased fluorescence of GRP78 after curli treatment in HLA-B27tg cells.

Colocalization of curli with local activation of GRP78 was observed at 1-h post-treatment (Figure 3). When we stained for GRP78 at 4 h post-treatment, we observed that curli-treated BMDMs from both HLA-B27tg (Supplementary Figure S2(b)) and C57BL/6 (Supplementary Figure S2(a)) had elevated GRP78 fluorescence, and curli-treated HLA-B27tg cells were significantly brighter than curli-treated C57BL/6 (Supplementary Figure S2(c)). At 24 h, curli-treated HLA-B27tg BMDMs maintained significantly higher GRP78 fluorescence intensity compared to non-treated (Figure 5(b,c)) and compared to curli-treated C57BL/6 (Figure 5(a,c)). Meanwhile, the GRP78 response faded in C57BL/6 at this timepoint (Figure 5(a,c)). We also observed that, at 4 h, there was only a subset of cells that had noticeable foci of GRP78 stain (Supplementary Figure S2), whereas at 24 h, most of the curli-treated HLA-B27tg cells had elevated GRP78 staining throughout the cell body (Figure 5(b)).

BMDMs isolated from (a) C57BL/6 or (b) HLA-B27tg cells were treated with 5 μg/mL curli for 24 h, then stained with anti-GRP78-FITC (green) and nuclei dyed with DAPI (blue). (c) Mean fluorescence intensity was measured on ImageJ on the green channel on 10 cells per image, four images per treatment, for a total of 40 cells per replicate. Background fluorescence was subtracted and MFI of curli-treated cells were normalized to the average untreated cells from the same mouse. Scale bars represent 20 μm. BMDMs isolated from *N* = 5 C57BL/6 and 6 HLA-B27tg mice. [Two-way ANOVA with multiple comparisons, ****= p < 0.0001].

### Elevated UPR in HLA-B27tg mice after acute STm infection

**Figure 6.**
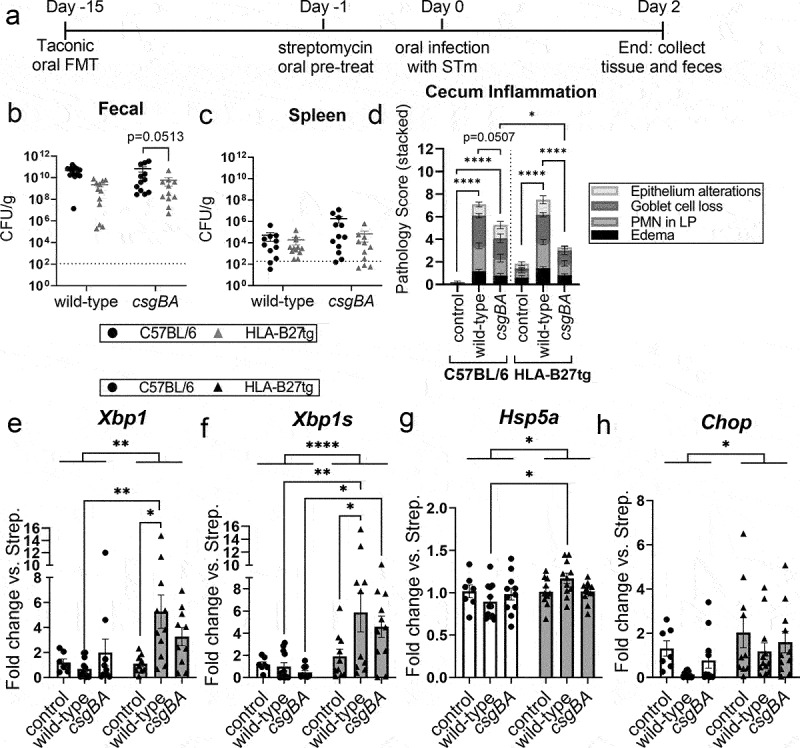
Elevated UPR after 48-h infection of HLA-B27tg with wild-type *Salmonella* Typhimurium.

HLA-B27tg mice do not spontaneously develop arthritis, however, it was demonstrated that a more inflammatory microbiome increases the inflammatory responses in these mice.^58^ Taconic mice have a microbiota that enhances Th17 response,^59^ which promotes autoimmunity. Our previous studies have demonstrated that mice with Taconic microbiota exhibit heightened inflammatory responses to curli.^22,60^ To confirm the *in* *vitro* results and examine UPR responses *in vivo*, age- and sex-matched HLA-B27tg and C57BL/6 mice from Jackson Laboratory underwent fecal microbiota transfer from donor C57BL/6 mice from Taconic farms (Figure 6(a)). Two weeks after microbiota transfer, mice were pre-treated with streptomycin then orally infected with wild-type or *csgBA* curli-mutant STm compared to uninfected controls. After 48 h, mice were sacrificed and STm colonization and inflammation were assessed (Figure 6(a)).

(a) C57BL/6 and HLA-B27tg mice underwent fecal microbiota transfer (FMT) from Taconic Farms mice. After 2 weeks, mice were pre-treated with streptomycin for 1 d, then infected with 10^8^ wild-type or *csgBA* (curli mutant) STm, or uninfected controls, for 2 d. (b) Fecal pellet and (c) spleen homogenates were plated and CFU/g calculated at 48 h post-infection; dotted line indicates the lower detection limit of CFU plating. (d) Inflammation in cecal tissue was scored by a blinded pathologist according to established criteria (Barthel et al. 2003) for epithelial alterations (integrity of the barrier vs. damage/erosion), loss of goblet cells in the epithelial layer, infiltration of polymorphonuclear (PMN) cells in the lamina propria (LP), and edema as scored by proportion of the diameter of the intestinal wall composed of submucosa. (e-h) RNA was extracted from cecal tissue and assessed by qRT-PCR for (e) total *Xbp1*, (f) spliced activated *Xbp1s*, (g) *Hsp5a*, and (h) *Chop* mRNA expression normalized to *Gapdh* and fold-change calculated vs. uninfected, strep-treated controls. *N* = 31 C57BL/6 and 33 HLA-B27tg mice in two replicate experiments. [(b-h) Two-way ANOVA with multiple comparisons, *= p < 0.05, **= p < 0.01, ****= p < 0.0001].

Samples were plated from the feces, spleen, and cecum to assess colonization. Although STm CFUs were lower in HLA-B27tg mice on average, these differences were not significant (Figure 6(b,c), Supplementary Figure S3(a)). Cecal inflammation was measured by blinded histopathological scoring using pre-established criteria.^61^ Wild-type STm-infected mice had increased cecal inflammation compared to uninfected controls, as expected, but there was no significant difference between C57BL/6 and HLA-B27tg wild-type STm-infected mice (Figure 6(d)). Uninfected controls had slightly higher pathology scores in the HLA-B27tg mice compared to C57BL/6, but this was not statistically significant. Curli-mutant (*csgBA*)-infected HLA-B27tg mice had significantly lower pathology than both the wild-type STm-infected HLA-B27tg and the *csgBA*-infected C57BL/6. However, pathology between wild-type STm-infected and *csgBA-*infected C57BL/6 mice was not significantly different (Figure 6(d)). Quantification of *Il1b* and *Il23* mRNA expression, both cytokines associated with Th17 activation, in the cecum homogenates of wild-type STm-infected HLA-B27tg mice revealed quite high *Il23 and Il1b* expression in a subset of mice, but these changes did not reach statistical significance, compared to C57BL/6 mice infected with wild-type STm (Supplementary Figure S3(b,c)).

When we examined the UPR in the cecum, expression of total *Xbp1* (Figure 6(e)) and spliced activated *Xbp1s* (Figure 6(f)) were both significantly elevated in wild-type STm-infected HLA-B27tg mice compared to C57BL/6 mice. The expression levels of these markers were overall significantly higher in all groups of HLA-B27tg mice over C57BL/6 mice. HLA-B27tg mice infected with the *csgBA* mutant had elevated levels of *Xbp1s* expression compared to *csgBA*-infected C57BL/6 mice (Figure 6(f)). However, *csgBA*-infected mice of either genotype failed to show significant UPR response compared to Strep.-treated uninfected controls (Figure 6(e-f)), indicating that curli is likely playing an important role in activating the UPR via the IRE1α/Xbp1 pathway *in vivo*. A pattern similar to *Xbp1/Xbp1s* was noted with *Hsp5a*, but the degree of change was low at a less than 1.5-fold change (Figure 6(g)). *Chop* expression showed no significant differences between infected and uninfected ceca; if anything, wild-type STm infection caused a slight decrease in *Chop* expression, which would be consistent with a recent study,^46^ and HLA-B27tg mice had significantly elevated *Chop* expression overall compared to C57BL/6 (Figure 6(h)). These results corroborate what we observed *in vitro*, that the presence of curli in the wild-type STm infection triggers elevated UPR response, but that this alone is not sufficient to dramatically alter inflammation.

### Colocalization of GRP78 and amyloid in macrophages in the lamina propria

To further examine the interaction between GRP78 and curli, cecal tissue sections from wild-type STm-infected, *csgBA-*infected, and uninfected HLA-B27tg mice were stained for GRP78, *Salmonella* O4 antigen, and curli using EBBA Biolight 680, a fluorescent bacterial amyloid stain that has demonstrated specificity for curli and not cellulose in STm biofilms.^62^

**Figure 7.**
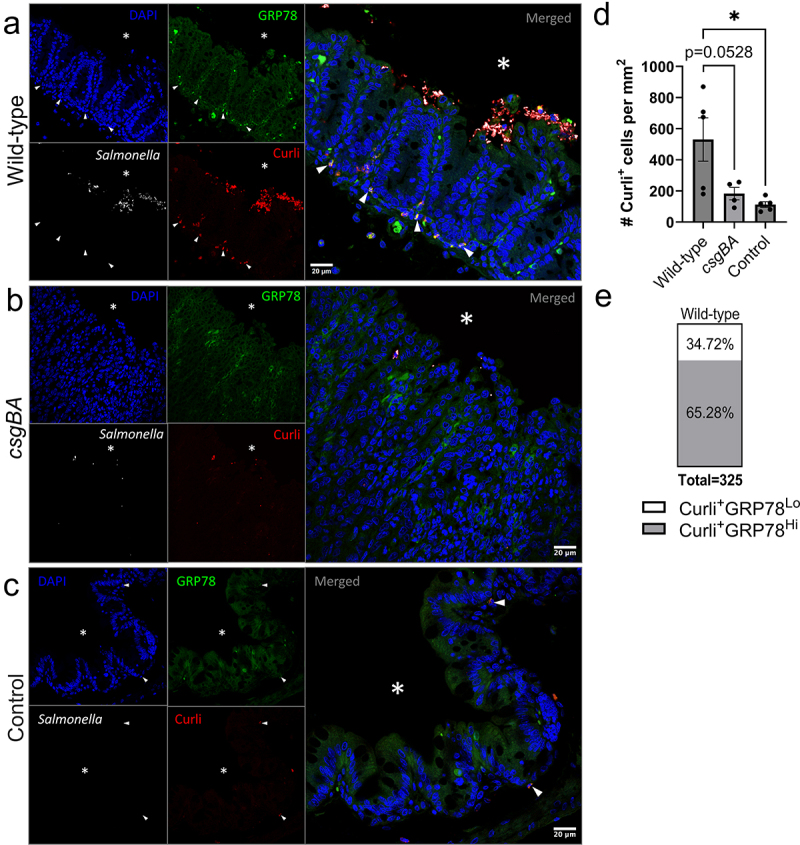
Colocalization of curli with GRP78 in ceca of infected mice.

*Salmonella* biofilms were visible in the cecal lumen of wild-type STm-infected mice: bacteria were clustered and surrounded by EBBA Biolight 680-stained curli, on the villi surface (Figure 7(a)), in addition to curli which appeared intracellular and not part of the biofilm in the lumen. Wild-type STm-infected mice demonstrated a significantly higher number of cells in the tissue where curli was internalized (Figure 7(d), Supplementary Figure S4(a)). GRP78 staining was also elevated in the tissues of infected mice but higher GRP78 fluorescence was only observed in a subset of cells, predominantly in the lamina propria, of wild-type STm-infected mice. 65.28% of the cells that contained curli showed high GRP78 (GRP78^Hi^) staining in the wild-type infected ceca, while 34.72% cells contained curli but they were low for GRP78 (GRP78^Lo^) (Figure 7(a-c,e)). Additionally, mean fluorescence intensity (MFI) of GRP78 had a significant positive correlation with the MFI of curli only in the wild-type STm-infected ceca and not in the *csgBA*-infected or Strep.-only controls (Supplementary Figure S4(b)).

Representative confocal microscopy images of cecal tissue from HLA-B27tg mice pre-treated with streptomycin then infected with (a) wild-type STm (*N* = 5), (b) *csgBA* STm (*N* = 4), or (c) uninfected, strep-treated controls (*N* = 5). Sections (5 μm) were stained for nuclei with DAPI (blue), anti-GRP78-FITC (green), *Salmonella* antiserum with anti-rabbit Rhodamine-X secondary (white), and for curli with EBBA Biolight 680, a bacterial amyloid dye (red). Scale bars represent 20 μm, 63X magnification. * = inside of the lumen; white arrow = selection of cells with colocalization. (d) Quantification of the number of Curli^+^ cells per mm^2^ counted in 3-4 fields per section per mouse, divided by the area of the field depicting tissue (not black background). (e) Mean fluorescence intensity (MFI) of all Curli^+^ cells was measured for both EBBA and anti-GRP78, then normalized to MFI of 5-10 Curli^−^GRP78^Lo^ (cells with basal levels of GRP78) sampled per field. GPR78^Lo^ was defined as within 2 standard deviations of the average MFI of Curli^−^GRP78^Lo^ sampled, to determine proportion of Curli^+^ cells that were either GRP78^Lo^ or GRP78^Hi^ [(d) One-way ANOVA with multiple comparisons, * = p < 0.05].

In uninfected control ceca, cells with GRP78 staining were less common, and so was EBBA Biolight 680 staining (Figure 7(c,d)), however neither stain was completely absent, because EBBA Biolight 680 may stain other amyloids from commensal bacteria, and because GRP78 is produced at basal levels. Additionally, streptomycin causes low-level inflammation,^61^ which may induce GRP78 in some cells even without infection. In mice infected with the curli mutant (*csgBA*), STm did not cluster into biofilm aggregates and intracellular curli staining was sparse (Figure 7(b,d)).

**Figure 8.**
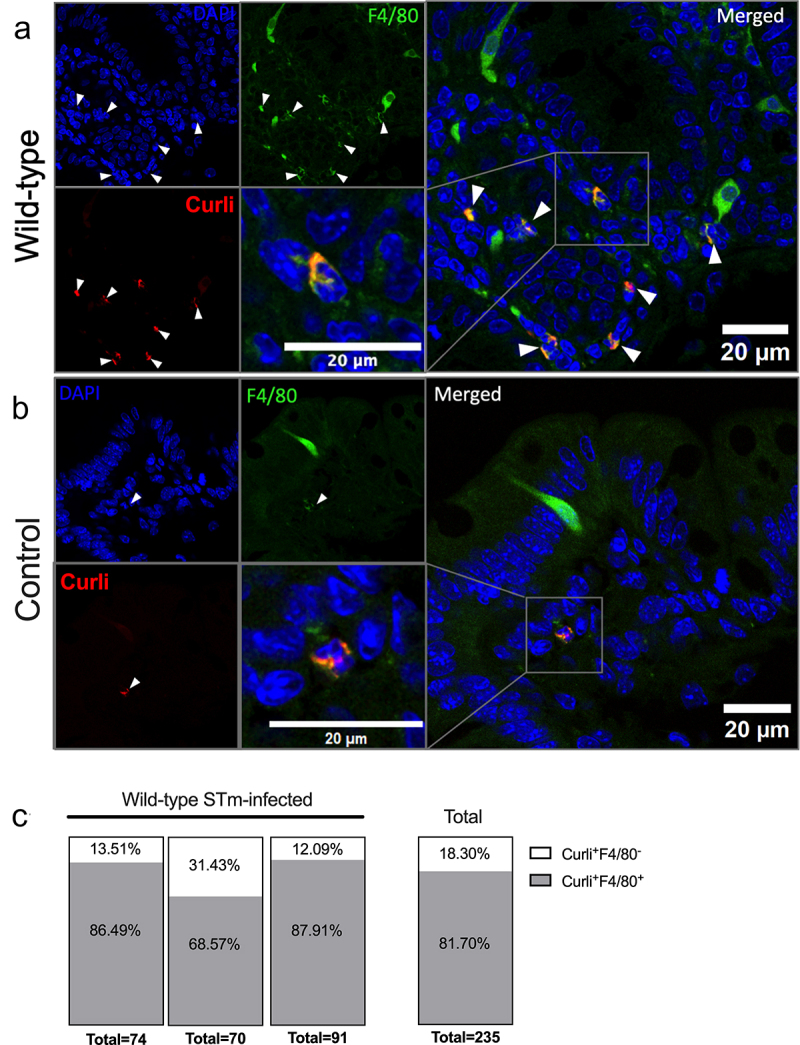
Colocalization of F4/80 with curli staining.

We hypothesized that the lamina propria cells that uptake curli that show increased GRP78 could be macrophages, so we stained cecum sections from the same experiment for F4/80, a macrophage marker, in addition to curli and GRP78 (Supplementary Figure S5). Strikingly, F4/80 colocalized with cells containing both curli and bright GRP78 staining, in the wild-type STm-infected HLA-B27tg mice (Supplementary Figure S5(a)), supporting the idea that the macrophages are a major cell type that contributes to UPR activation and curli uptake. Again, the uninfected ceca showed less GRP78 and curli staining, as well as less macrophage staining (Supplementary Figure S5(b)), which is consistent with previous studies that showed that STm infection leads to the recruitment of macrophages to the gut.^63^ These findings were further confirmed using a different fluorophore for F4/80, which has a spectrum farther from the EBBA Biolight 680 used to stain curli, with additional steps to reduce autofluorescence and enhance antigen retrieval for optimized signal-to-noise ratio (Figure 8, Supplementary Methods). Confirming the previous result, F4/80 staining colocalized with the curli staining in the tissue sections from wild-type STm-infected HLA-B27tg mice: 81.7% of curli-containing cells were also positive for F4/80 (F4/80^+^) in wild-type STm-infected HLA-B27tg ceca (Figure 8(a,c), Supplementary Figure S6(a,b)). Uninfected controls had rare visible macrophages and intracellular curli staining (Figure 8b). These results suggest that the majority of cells that uptake curli in the lamina propria are macrophages. However, further investigation is needed to identify the remaining 18.3% of cells that uptake curli, which may include other phagocytes such as dendritic cells or neutrophils.

Representative confocal microscopy images of cecal tissue from HLA-B27tg mice pre-treated with streptomycin then infected with (a) wild-type STm or (b) uninfected, strep-treated controls (*N* = 3 HLA-B27tg mice each). Sections (5 μm) were stained for nuclei with DAPI (blue), anti-F4/80-AlexaFluor488 macrophage marker (green), and for curli with EBBA Biolight 680 amyloid marker (red). (c) All Curli^+^ cells were selected and counted in 9 fields per cecal section/mouse and mean fluorescence intensity measured on both EBBA and anti-F4/80, then normalized to MFI of 5-10 double negative cells sampled per field. F4/80^−^ were defined as within 3 standard deviations of the average MFI of double negative cells sampled. Scale bars represent 20 μm, 63X magnification. White arrow = cells with colocalization.

## Discussion

During infection, the inflammatory response, immune cell recruitment, and activation of virulence mechanisms by pathogens create a challenging environment in the intestinal tract. In response to such stresses, host cells in the gut initiate mechanisms to support cellular function and adapt to changing environmental conditions. One such adaptive response is the activation of the unfolded protein response triggered by ER stress. Intriguingly, genetic factors like HLA-B27, a risk factor for ReA following *Salmonella* infection, have been associated with elevated levels of UPR and ER stress,^2,24,25^ which may contribute to the development of dysregulated inflammation and autoimmunity. However, the mechanisms that lead patients with gastroenteritis to develop the autoimmune condition ReA are not fully understood.

Recently, we identified *Salmonella* biofilms and the abundant biofilm matrix component, curli, as well as its translocation from the intestinal tract, as a potential key factor in the pathogenesis of ReA.^8,16,23,60,64^ Here, we demonstrate for the first time a mechanistic link between curli, UPR-induced inflammation, and HLA-B27. We show that curli triggers the activation of UPR, particularly in HLA-B27tg cells and mice. However, this increased UPR does not dramatically alter cytokine production induced by curli, *in vitro* (Figures 1–5) or *in vivo* (Figures 6–8). However, by using inhibitors specific to different branches of the UPR pathway, we demonstrate that the IRE1α pathway, in particular, *is* involved in curli-induced IL-6 production and ER stress responses (Figure 2), despite the lack of *differential* cytokine response to curli in HLA-B27tg vs. wild-type macrophages.

Previous studies have shown that curli stimulates the TLR2/1 heterocomplex while the DNA associated with curli in the biofilm matrix activates TLR9.^18,23^ It is established that TLR stimulation feeds into the UPR through IRE1α.^41,42^ Therefore, it is possible that activation of TLRs by curli may contribute to the UPR in response to curli. However, it is also possible that the amyloid structure of curli fibers would allow curli to directly activate the UPR, via mechanisms similar to those observed with α-synuclein and amyloid-β, human pathogenic amyloids that provoke neuroinflammation, in large part due to their activation of ER stress and the UPR.^54–56^ Notably, α-synuclein aggregates added exogenously to cells directly interact with GRP78 and with the SERCA pump on the cytosolic side of the ER to activate the UPR.^54–56^ It has previously been demonstrated that curli escapes the endosome into the cytosol of macrophages following phagocytosis.^23^ Here, we observed colocalization of curli with locally elevated GRP78 inside the cytosol of macrophages *in vitro* (Figure 3). Importantly, synthetic CsgA, α-synuclein and amyloid-β fibrils added exogenously to BMDMs all colocalized with GRP78 (Figure 4), confirming that both bacterial and host amyloid aggregates interact with the GRP78 leading to the activation of the UPR. Similar results were observed with curli and GRP78 in lamina propria macrophages *in vivo* following STm infection (Figures 7–8). In fact, this is the first study to report the uptake of curli by lamina propria macrophages, past the gut epithelial barrier. Therefore, macrophages are likely critical players of immune responses to curli including the development of autoimmunity.

Despite the elevated UPR after curli treatment *in vitro* (Figure 1) or *in vivo* (Figure 6) in HLA-B27tg mice, the elevated UPR alone did not significantly increase the pro-inflammatory cytokine response or overall cecum inflammation *in vitro* or *in vivo* in HLA-B27tg mice compared to C57BL/6. Future studies are needed to further address the role of curli-induced UPR responses in autoimmunity. As C57BL/6 mice are resistant to developing arthritis,^65^ other models might be necessary to further study these responses. Future studies with HLA-B27tg rats, which are more susceptible to autoimmunity and better represent inflammatory disease seen in humans,^66^ may be used to further investigate the role of curli in *Salmonella*-induced ReA.

In this study, curli taken up by macrophages was located in the cytoplasm co-localized with GRP78. While GRP78 primarily localizes to the ER membrane, during cellular stress it has also been observed in endosomes, in autophagosomes that derived from the ER membrane,^67^ or there is even a cytosolic isoform, GRP78va, thought to be involved during elevated stress and autophagy.^68–70^ The UPR is known to activate autophagy and other degradation pathways^71^ and HLA-B27 has been implicated in dysregulation of degradation.^72,73^ Mutations in autophagy and the autophagy-related NOD2 pathway have been linked with IBD and Crohn’s disease,^74,75^ which are associated with the very same IL-23/Th17 inflammation and HLA-B27 genetic risk as reactive arthritis; in fact, all three diseases fall under the HLA-B27-associated spondylarthritis umbrella.^74^ Autophagy and NOD pathways have also been implicated in host–cell defense against intracellular *Salmonella* infection.^45,46^ The exact cytosolic compartment containing the intracellular curli and elevated GRP78, which may in fact be the autophagosome and not the ER, must still be determined with more specific stains and assays.

Here, we observed increased expression of GRP78 in HLA-B27tg macrophages at 24 h after curli treatment, while the response in C57BL/6 cells appeared to fade between 4 and 24 h (Figure 5, Supplementary Figure S2). This suggests a longevity of activation, or longevity of the curli itself, inside of HLA-B27+ macrophages, possibly due to impaired degradation.^72,73,76^ So, perhaps this dysfunction of degradation pathways for aggregate proteins, such as autophagy, due to HLA-B27 misfolding, contributes to impaired curli degradation, allowing curli to persistently provoke inflammation.

Additionally, it is unlikely that curli alone contributes to UPR-induced inflammation associated with HLA-B27 and autoimmunity. Other studies cite intracellular *Salmonella* replication and the type 3 secretion system synergizing with HLA-B27 misfolding to exacerbate inflammation.^50,76^ Furthermore, the PERK/CHOP branch of UPR has been shown to have an inverse response to STm infection compared to the IRE1α and ATF6 branches, and implicated host iNOS expression and nitrate bioavailability for STm colonization and inflammation in the gut.^46^ How curli is degraded within cells and how it, and possibly other *Salmonella* factors, interacts with the UPR, and perhaps autophagy, are all interesting questions still in need of elucidating and may shed light on a novel interaction between *Salmonella* infection, the UPR, and HLA-B27 in the stress response that eventually leads to reactive arthritis.

## Materials and methods

### Animal models and ethics statement

C57BL/6 and HLA-B27tg were obtained from Jackson Laboratory, and C57BL/6 from Taconic Farms as donors for fecal microbiota transfer. Experiments were performed in a BSL2 facility under protocols (#4868) approved by AALAC-accredited Temple University Lewis Katz School of Medicine, Institutional Animal Care and Use Committee, on file with the NIH Office for the Protection of Research Risks, in accordance with USDA and PHS Policy on Human Care and Use of Laboratory Animal Welfare. Mice were age- and sex-matched within each replicate experiment: infections were performed on mice 6–8 weeks old, and BMDMs were isolated from mice 10–30 weeks old. Analyses found no significant differences between male and female mice, so the data were pooled.

### Bacterial strains and cultures

*Salmonella enterica* serovar Typhimurium IR715, our wild-type strain derived from the ATCC 14,028 strain,^77^ and its isogenic *csgBA* unmarked curli mutant,^22^ were grown in Luria-Bertani (LB) broth supplemented with 50 μg/mL nalidixic acid at 37°C. The isogenic *msbB* (LPS) mutant was previously described^78^ and grown in LB broth with 100 μg/mL kanamycin at 37°C.

### Curli purification

Curli aggregates were purified from the *msbB* mutant as previously described^52^ (Supplementary Methods). Curli purified in this manner still contains approximately 200–400 ng complexed DNA per mg of purified curli, despite DNAse treatment steps, due to curli’s ability to protect DNA from digestion.^9,23,52^

### Bone marrow-derived macrophages and treatments

BMDMs from C57BL/6 and HLA-B27tg mice were isolated as previously described^19^ with some modifications (Supplementary Methods). For qRT-PCR and ELISA analyses, BMDMs were seeded in 24-well tissue culture plates at 5 × 10^5^ cells/well in RPMI with 10% FBS. After 24 h, BMDMs were treated with 2.5 μg/mL curli, 100 ng/mL LPS-SM Ultrapure from *Salmonella* (InvivoGen, #tlrl-smlps), or sterile PBS for untreated controls. For inhibitor experiments, BMDMs were pre-treated with 1 μM (1X) or 2 μM (2X) KIRA6 (MedChemExpress), 50 μM (1X) or 100 μM (2X) STF-08310 (MedChemExpress), 500 nM (1X) or 1 μM (2X) GSK2656157 (MedChemExpress), 200 μM TUDCA (MedChemExpress),^38^ and/or the appropriate volume of DMSO for vehicle controls, keeping the total volume of DMSO plus inhibitor consistent for all treatments. After 30 min, BMDMs were treated with 2.5 μg/mL curli. After treatment, cells were allowed to incubate at 37°C for 24 h before supernatant was harvested for ELISA or MSD analyses and cells were incubated in TRI-Reagent (Molecular Research Center, TR 118) and then stored at −80°C for later RNA extraction and qRT-PCR analyses.

### RNA extraction

For BMDMs, RNA was extracted from cells suspended in TRI-Reagent according to the manufacturer’s instructions (Molecular Research Center, Inc.) and as previously described^79^ with the following modifications: samples underwent two subsequent chloroform extraction steps to decrease phenol contamination.^80^ Then, the aqueous layer was mixed with isopropanol and precipitated at −80°C for 1 h to overnight. Nucleic acids were pelleted then washed twice with 70% ethanol to increase quality of RNA^80^ and then dried. Pellets were resuspended in the appropriate volume of nuclease-free water and underwent DNAse digestion with the TURBO^TM^ DNAse kit (Invitrogen by Thermo Fisher, 2238 G) according to the manufacturer’s instructions.

For mouse tissue, a small piece of cecum approximately 2 mm dimensions was collected from infected mice and stored at −80°C until processing. RNA was extracted using the FastRNA Green Kit (MP Bio 116045-050) and homogenized using the FastPrep-24 shaker (MP Bio), following the manufacturer’s protocol. RNA concentration and quality from either extraction were measured on a NanoDrop One (Thermo).

### cDNA and qRT-PCR

RNA was reverse transcribed into cDNA as previously described.^79^ Briefly, RNA from each sample was individually diluted to 0.1 μg/gene in nuclease-free water added to a total of 2.075 μl/gene. Then, master mixes of MgCl_2_ (5.5 mM, Thermo Scientific), RT Buffer without MgCl_2_ (1X, Applied Biosystems, N100025924), dNTP Mix (2 mM, Applied Biosystems 362,275), Random Hexamer Primers (5 ng/μl, Thermo Scientific, SO142), RNAse Inhibitor (0.4 U/μl, Applied Biosystems, N8080119), and Multiscribe Reverse Transcriptase (1 U/μl, Applied Biosystems 4,308,228) was added, to a total of 2.925 μl/sample/gene to the diluted RNA. Then, an Applied Biosystems MiniAmp thermocycler was used to reverse transcribe cDNA (Step 1: 25°C for 10 min, Step 2: 48°C for 30 min, Step 3: 95°C for 5 min, Step 4: 4°C hold).

For qPCR, as previously described,^79^ PowerUp^TM^ SYBR^TM^ Green Master Mix (Applied Biosystems by Thermo Fisher, A25742) was mixed with appropriate primers at 10 μM, from Integrated DNA Technologies, and loaded into duplicate wells of a MicroAmp qPCR plate. qPCR was performed with an Applied Biosciences StepOne Plus Real Time PCR system. Relative transcript levels were determined by ΔC_T_ normalized to *Gapdh*, and the fold-change was calculated versus non-treated controls.

UPR markers were measured by qRT-PCR for mouse *Hsp5a* (Forward: 5’- GAGCGTCTGATTGGCGATGC, Reverse: 5’-TTCCAAGTGCGTCCGATGAGG,^43^); total *Xbp1* (Forward: 5’-GAGTCCGCAGCAGGTG, Reverse: 5’-GTGTCAGAGTCCATGGGA,^43^); spliced *Xbp1* (Forward: 5’-AGCTTTTACGGGAGAGAAAACTCA, Reverse: 5’-GCCTGCACCTGCTGCG,^81^); and *Chop* (Forward: 5’- CTGGAAGCCTGGTATGAGGAT, Reverse: 5’-CAGGGTCAAGAGTAGTGAAGGT,^43^). Cytokine mRNA expression was measured using the primers for mouse *Il6* (Forward: 5’- TCCAGAAACCGCTATGAAGTTCC, Reverse: 5’-CACCAGCATCAGTCCCAAGAAG,^82^); *Il1b* (Harvard Primer Bank ID 6680415a1), and *Il23* (Forward: 5’-TGTGCCTAGGAGTAGCAGTCCTGA, Reverse: 5’-TTGGCGGATCCTTTGCAAGCAGAA,^83^). All mRNA was normalized against mouse *Gapdh* (Forward: 5’-AGGTCGGTGTGAACGGATTTG, Reverse: 5’-TGTAGACCATGTAGTTGAGGTCA, Harvard Primer Bank ID 6679937a1).

### Cytokine quantification

Supernatants from BMDM assays were stored at −20°C for later ELISA analysis. IL-6 ELISA was performed on supernatants diluted to 1:40 following the manufacturer’s instructions for the Mouse IL-6 Uncoated ELISA Kit (Invitrogen, Cat. #88-7064). MSD analysis was performed on supernatants diluted 1:10 or 1:20 following the manufacturer’s instructions for the U-PLEX Custom Biomarker Group 1 (ms) Assay (Meso Scale Discovery, K15069M-1) with U-PLEX Mouse GM-CSF, IFN-β, IL-1β, IL-6, IL-10, IL-23, and TNF-α.

### BMDM staining and confocal microscopy

BMDMs were seeded at 2 × 10^6^ cells/well in 500 μl of BMDM media in 24-well tissue culture plates, prepared with circular coverslips (Supplementary Methods). After 24 h, BMDMs were treated with either 5 μg/mL of curli for 4 h or 24 h, or 20 μg/mL of curli stained with Congo Red (curli-CR) for 1 h. Curli was stained with Congo Red as previously described,^23^ briefly: 1 mg/mL of curli stock was incubated for 15 min in the dark with 50 μg/mL Congo Red (Sigma, C6767), washed five times with sterile PBS, and diluted to 500 μg/mL before treating cells the same day.

To test other amyloids, we utilized synthetic CsgA R4–5 (BioSynthesis), which consists of the 4^th^ and 5^th^ repeats of the CsgA monomer, which is capable of fibrillization and TLR activation;^23^ α-synuclein purified from *E. coli* BL21 (DE3) transformed with pT7-7 plasmid for overexpression of α-synuclein monomer, purified following a previously described protocol;^84^ or amyloid-β-42, synthesized by Peptide 2.0 and monomers isolated as previously described.^85^ Monomers of each were diluted to 500 µg/mL in potassium phosphate buffer (28.9 mM KH_2_PO_4_ and 21.1 mM of K_2_HPO_4_, total of 50 mM), and left to fibrillize at 37°C for 48 h. Then, fibrillized csgA, α-synuclein, and amyloid- β were stained following the same protocol as above and used to treat BMDMs. After treatment, cells were fixed, permeabilized, stained for GRP78 and DAPI (Supplementary Methods, Supplementary Table S1), and then mounted and imaged on a confocal microscope, processed on ImageJ2 (Fiji, Version 2.14.0), and mean fluorescence intensity measured (Supplementary Methods).

### Streptomycin pre-treatment and acute infection

Mice were colonized with Taconic microbiota through fecal microbiota transfer to 2 weeks (Supplementary Methods) and then orally gavaged with 100 μl of 200 mg/mL streptomycin sulfate salt (Sigma, S9137-100 G) filter sterilized using 0.22 μm Millipore Express Plus Membrane sterile disposable vacuum filtration system (Millipore, SCGP00525). After 24 h, mice were orally gavaged with 100 μl of LB broth containing 10^8^ bacteria, diluted from an overnight culture grown for approximately 18 h at 37°C; uninfected controls were orally gavaged with LB broth alone. Inoculum concentration was confirmed by diluting and counting CFU on LB agar supplemented with nalidixic acid. Mice were sacrificed 48 h after infection and tissue samples were collected.

### Quantification of STm infection in vivo

Forty-eight hours post-infection, fecal, spleen, and cecal tissue samples were collected and weighed in sterile PBS. Feces and spleen were homogenized immediately, cecal tissue was washed three times with sterile PBS, with vortexing, incubated for 1.5 h in sterile PBS containing 100 μg/mL gentamycin sulfate (Corning, 30-005-CR) at 37°C, then washed thrice again with sterile PBS, before finally homogenizing. Serial dilutions of sample homogenates were prepared in sterile PBS and plated on LB agar supplemented with nalidixic acid and then grown overnight at 37°C. CFU were counted, with a countable range of 30–300 colonies, and CFU per gram of feces or tissue was calculated for each mouse.

### Histopathological scoring of the murine cecum

Tissue from the tips of the ceca of infected mice was fixed for 48 h in 10% formalin and then transferred into 70% ethanol for storage. Tissue was embedded in paraffin at Fox Chase Cancer Center Histopathology Core Facility, and 5 μm sections of the tissue were stained with hematoxylin and eosin. Samples were blinded and evaluated by an experienced veterinary pathologist according to the criteria outlined previously.^61^

### Cecal tissue immunohistochemistry

Slides were sectioned (5 μm) from the same paraffin-embedded cecal tissues as for histopathological scoring. Tissue was deparaffinized and stained with the appropriate antibody cocktails (Supplementary Methods, Supplementary Table S1). Tissue was imaged as with BMDMs on the confocal microscope using sequential scans at the appropriate spectra for each antibody used (Supplementary Table S1). Images were processed and quantified in ImageJ2 (Supplementary Methods, Supplementary Figure S4 & S6).

### Statistical analysis

Data were analyzed on GraphPad Prism [Version 9.3.1 (350)] using the appropriate statistical tests: two-way ANOVA with multiple comparisons test for qPCR, ELISA/MSD, CFU, and histopathology scores (Figures 1 & 6, Supplementary Figure S3); one-way ANOVA with multiple comparisons test for MFI and cell counts (Figures 5 & 7, Supplementary Figure S2); simple linear regression and probability of significantly non-zero slope for correlation data (Figure 1, Supplementary Figure S4); or one sample t-test for ELISA or qPCR data from inhibitor treatments normalized to DMSO+curli group from the same batch of BMDMs (Figure 2).

## Abbreviations

STm: *Salmonella enterica* serovar Typhimurium
ReA: reactive arthritis
UPR: unfolded protein response
HLA-B27: human leukocyte antigen-B27
HLA-B27tg: HLA-B27 transgenic mice
BMDMs: bone marrow-derived macrophages
eDNA: extracellular DNA
TLR: toll-like receptor

## Supplementary Material

Supplemental Material
