# Supplementary material for: Bacterial amyloid curli activates the host unfolded protein response via IRE1α in the presence of HLA-B27
Source: Gut Microbes. 2024 Aug 27;16(1):2392877. doi: 10.1080/19490976.2024.2392877 (PMC11352795; doi:10.1080/19490976.2024.2392877)
Supplement: Supplemental Material [file KGMI_A_2392877_SM2167.zip › Supplementary_Materials_2nd_REVISIONS_v13_no_tracking.docx]

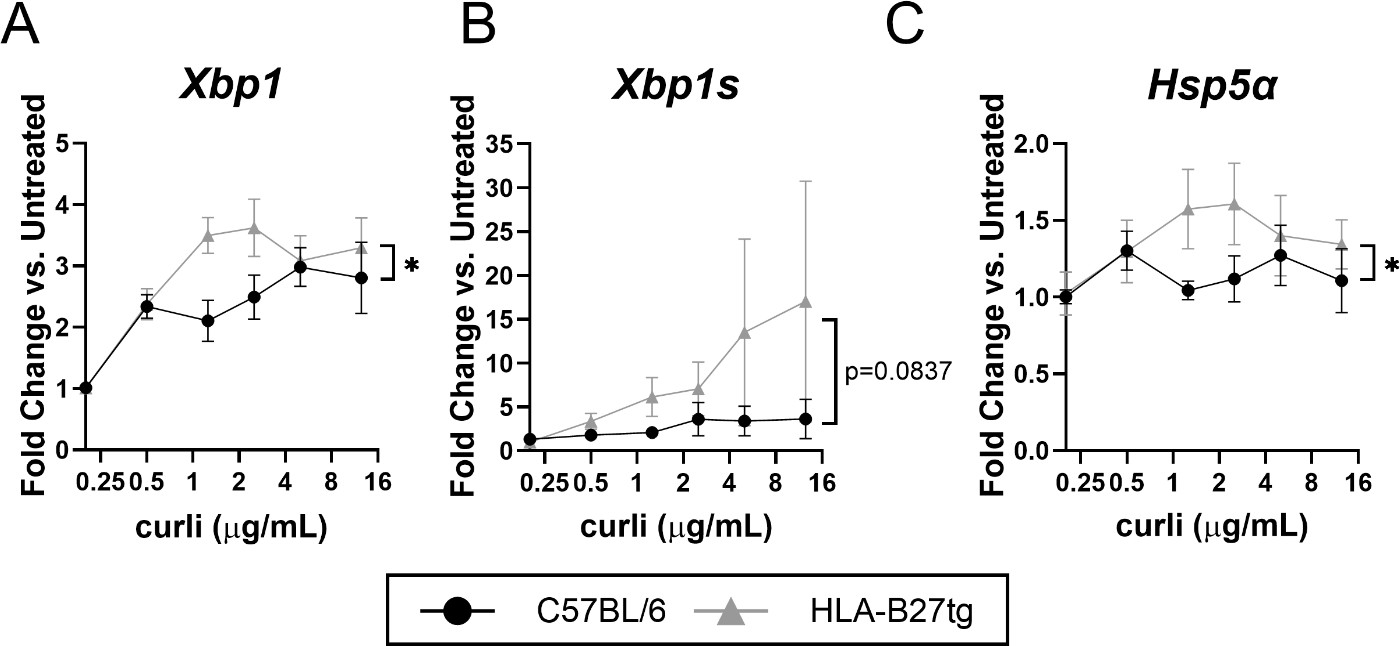


**Supplementary Figure 1. Curli dose curve and pro-inflammatory cytokines.** BMDMs were cultured from C57BL/6 (black, circles) or HLA-B27tg (gray, triangles) mice and treated with increasing doses of curli from 0.5 μg/mL to 12.5 μg/mL. qRT-PCR on RNA extracted from BMDMs looking at UPR markers: (A) total *Xbp1*, (B) spliced activated *Xbp1s*, or (C) *Hsp5a*, normalized to untreated controls. N = 4 HLA-B27tg mice and 4 C57BL/6 mice cultured in parallel. [Two-way ANOVA with multiple comparisons, * = p<0.05]


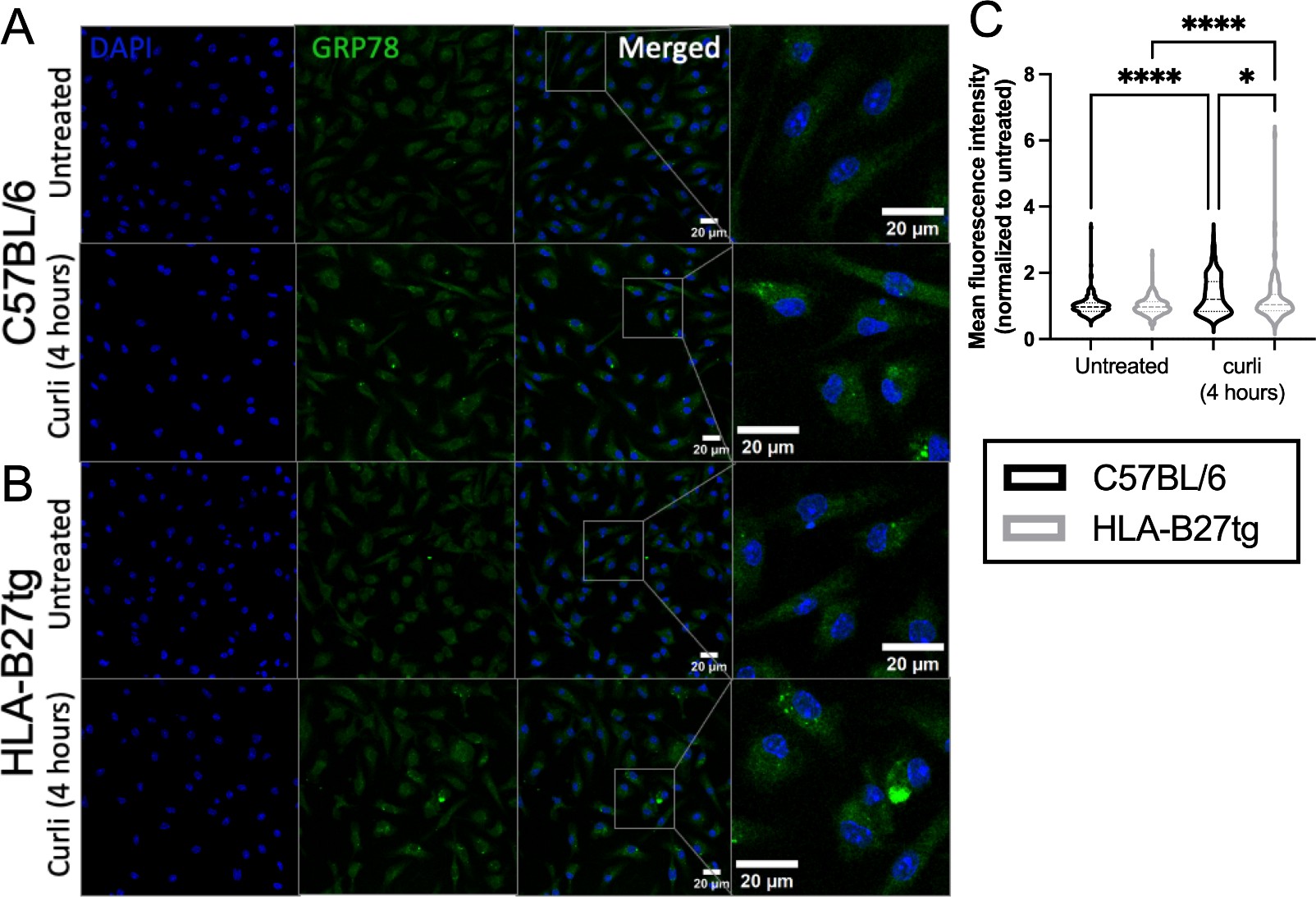


**Supplementary Figure 2. GRP78 staining 4 hours post-curli treatment.** BMDMs isolated from

(A) C57BL/6 or (B) HLA-B27tg cells were treated with 5 μg/mL curli for 4 hours, then stained with anti- GRP78-FITC (green) and nuclei dyed with DAPI (blue). (C) Mean fluorescence intensity was measured on ImageJ on the green channel on 10 cells per image, 4 images per treatment, for a total of 40 cells per replicate. Background fluorescence was subtracted and MFI of curli-treated cells were normalized to the average untreated cells from the same mouse. Scale bars represent 20μm, 63X magnification. BMDMs isolated from N = 3 C57BL/6 and 3 HLA-B27tg mice. [(C) Two-way ANOVA with multiple comparisons, * = p<0.05, **** = p<0.0001)


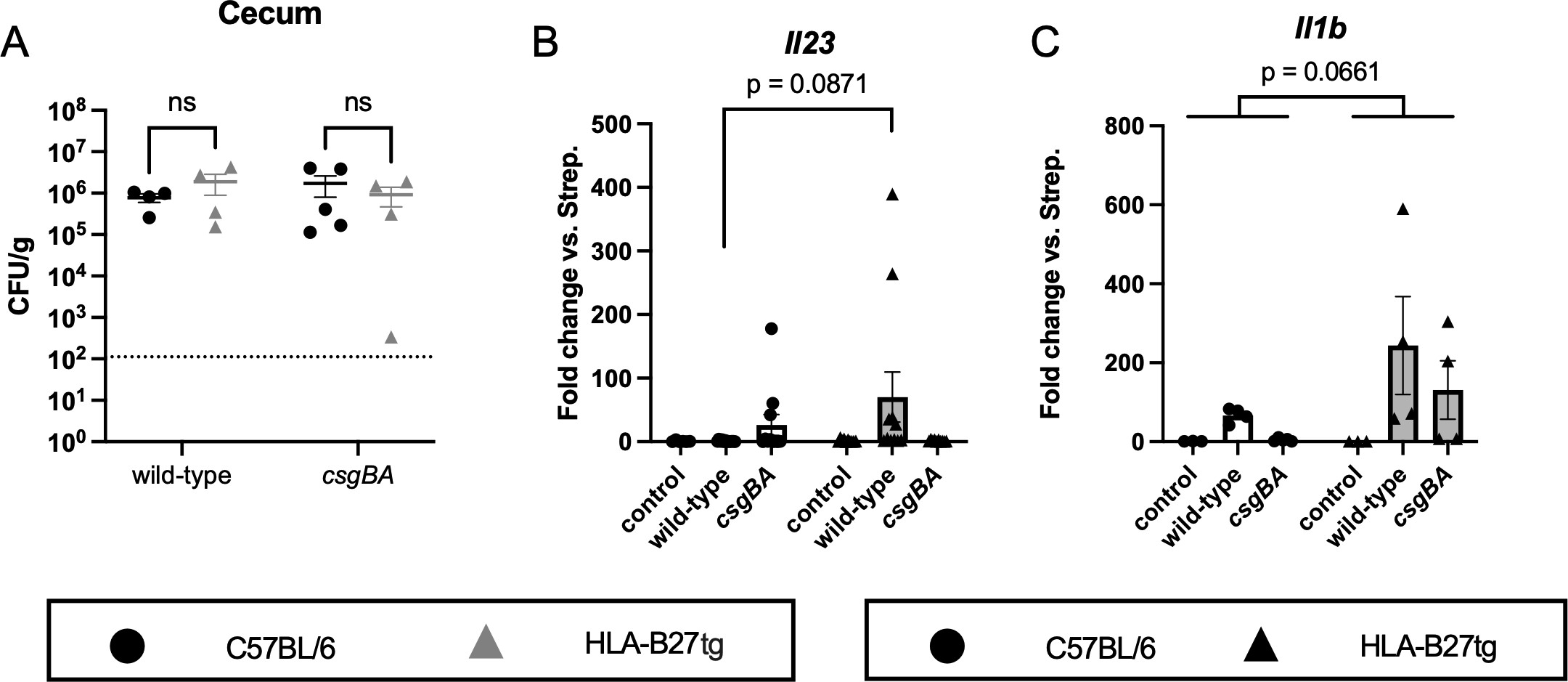


**Supplementary Figure 3. 48h infection of HLA-B27tg vs. C57BL/6.** (A) Cecum homogenate was plated and CFU/g calculated (N = 9 C57BL/6, 8 HLA-B27tg); dotted line indicates the lower detection limit of CFU plating. (B-C) RNA was extracted from cecal tissue and qRT-PCR determined mRNA expression of (B) *Il23* (N = 31 C57BL/6, 33 HLA-B27tg) and (C) *Il1b* (N = 12 C57BL/6, 11 HLA-B27tg) normalized to *Gapdh* and fold change calculated against uninfected, streptomycin-treated control. [Two-way ANOVA with multiple comparisons, ns = not significant]


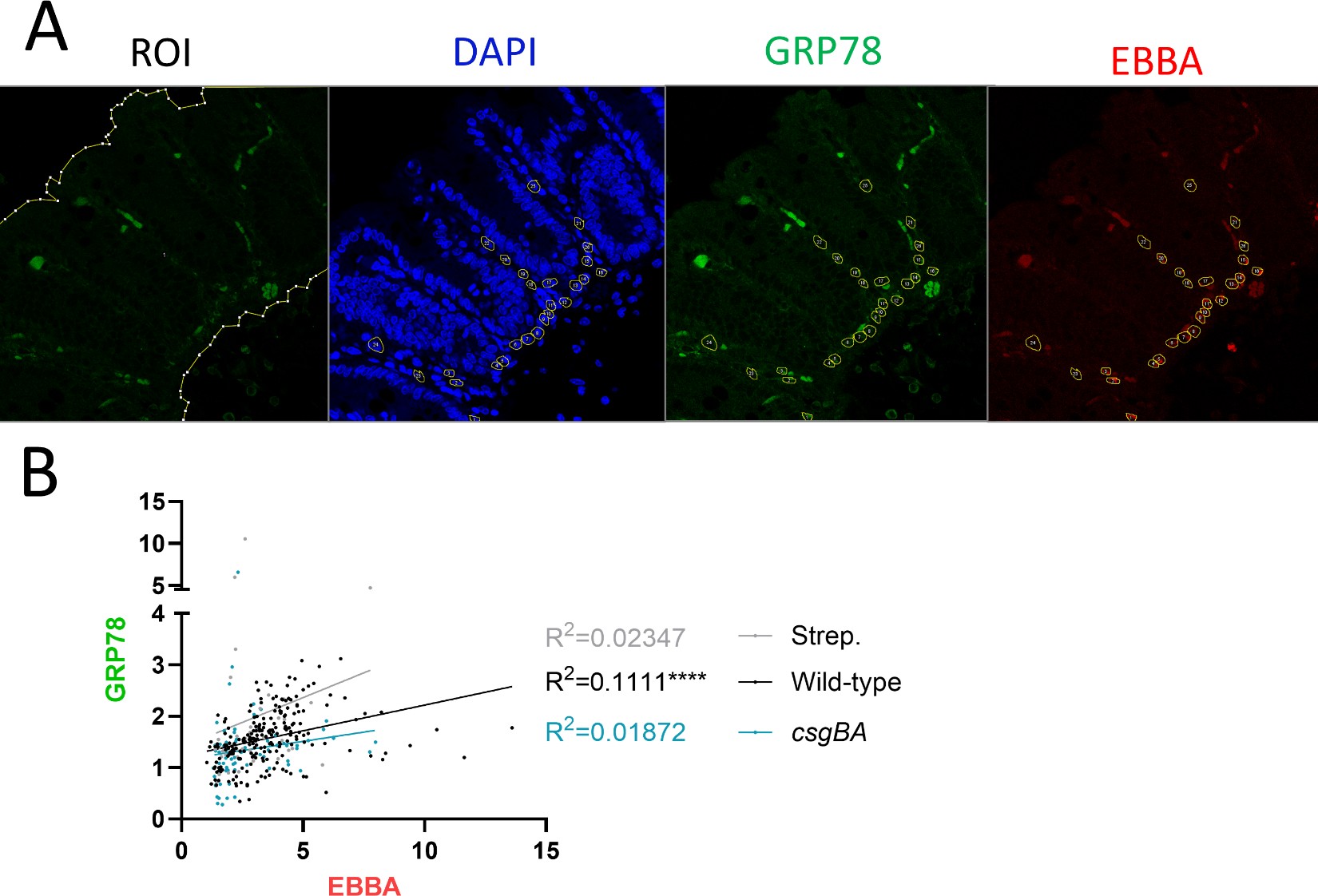


**Supplementary Figure 4. Quantification of GRP78 and EBBA staining.** (A) Example screenshots from ImageJ2 of the selection, first, of the region of interest (ROI) which included only the tissue, for quantification of the area in which cells were counted. Then all EBBA+ cells were selected and counted, followed by a sampling of 5-10 EBBA^-^GRP78^Lo^ (cells with baseline low level GPR78 staining) from the lamina propria while avoiding cells that looked like epithelium (lined up flat in a neat row) or red blood cells (no nuclei, brighter green autofluorescence), for normalization of mean fluorescence intensity (MFI) within each field measured. (B) EBBA MFI vs. GRP78 MFI distribution (normalized to average MFI of EBBA^-^GRP78^Lo^. Tested significance of non-zero slope (F-test) (**** = p<0.0001)


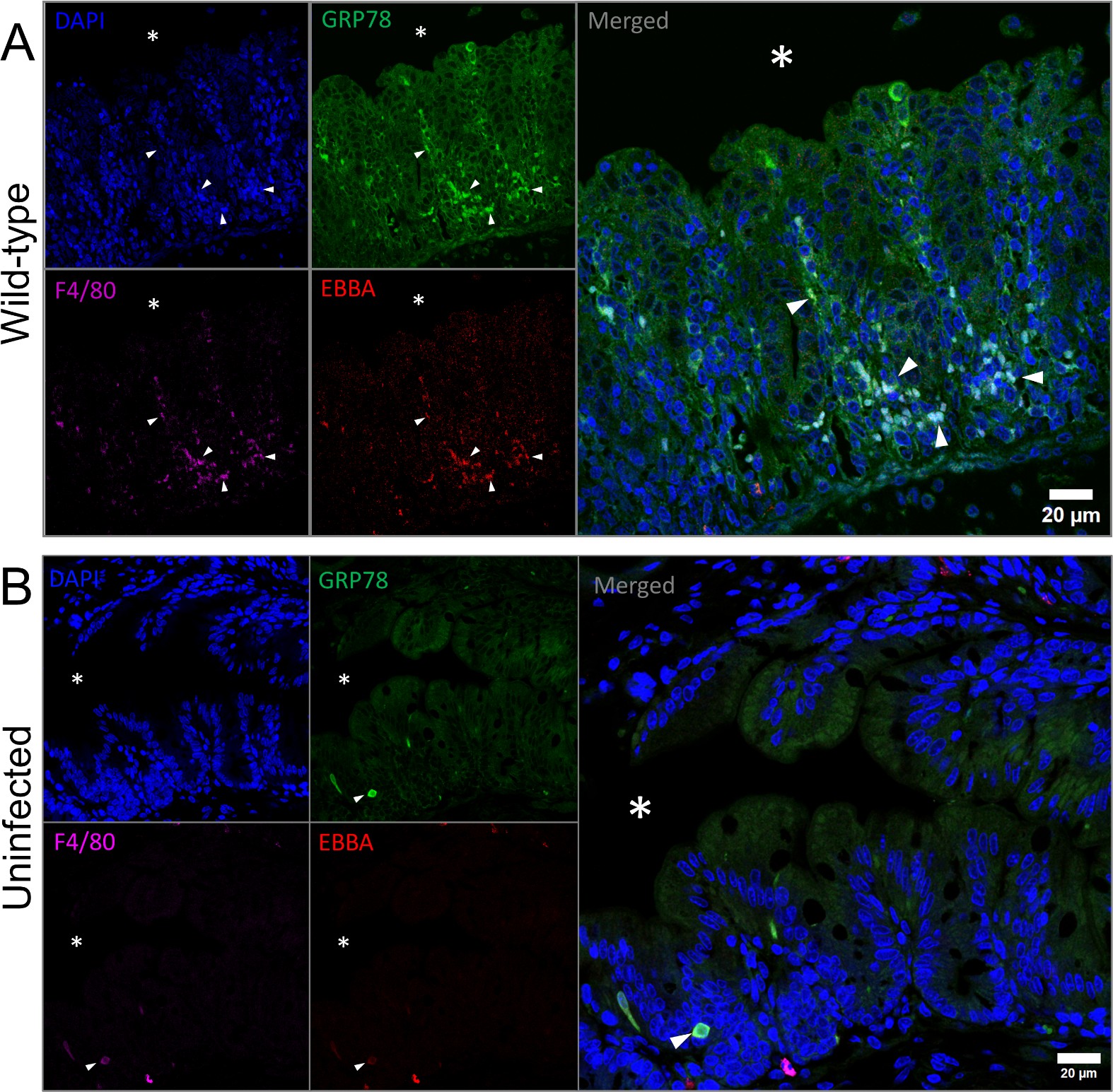


**Supplementary Figure 5. Colocalization of F4/80 with GRP78 and EBBA staining.** Representative confocal microscopy images of cecal tissue from HLA-B27tg mice infected with (A) wild-type STm or (B) uninfected, strep-treated controls (N = 4 HLA-B27tg mice). Sections (5 μm) were stained for nuclei with DAPI (blue), anti-GRP78-FITC (green), anti-F4/80-PE macrophage marker (magenta), and EBBA Biolight 680 (red). Scale bars represent 20μm, 63X magnification. * = inside of the lumen; white arrow = selection of cells with colocalization.


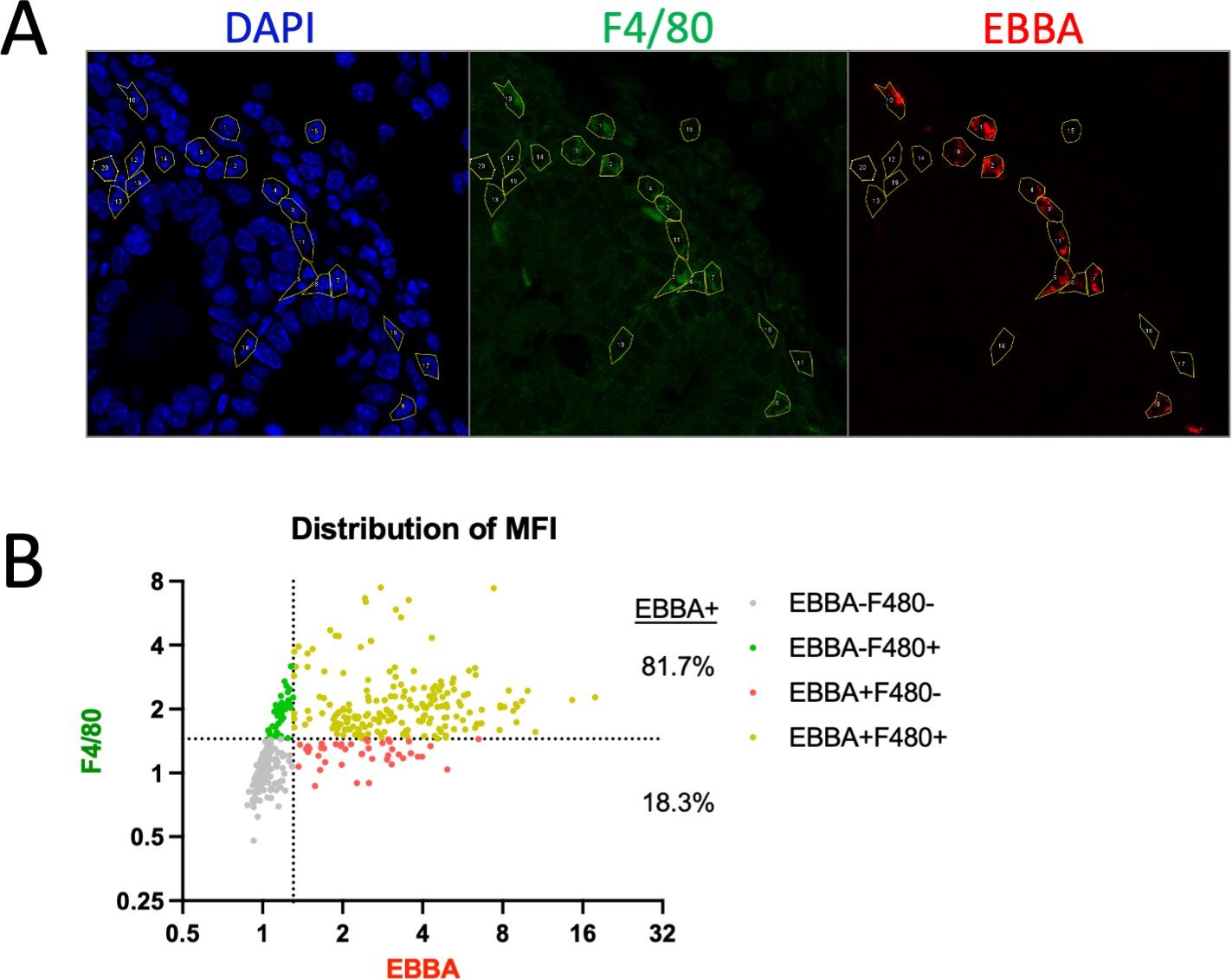


**Supplementary Figure 6. Quantification of F4/80 staining in EBBA+ cells.** (A) Example of selection of all EBBA^+^ and sampling of 5-10 double negative cells per field for quantification of mean fluorescence intensity (MFI) on ImageJ2 and subsequent categorization for EBBA^+^ cell counting.

Double negatives were selected from lamina propria cells while avoiding cells that looked like epithelium (lined up flat in a neat row) or red blood cells (no nuclei, brighter autofluorescence). (B) EBBA MFI vs. F4/80 MFI distribution (normalized to average MFI of double negatives), including sampling of double negative (gray) for normalization and some EBBA^-^F4/80^+^ (green) cells for defining F4/80^+^ vs. F4/80^-^. Dotted lines indicate MFI cutoffs for categorization.

# Supplementary Table 1.

| **Antibody/Dye** | **Dilution** | **Source** | **Laser Settings** |
| --- | --- | --- | --- |
| DAPI | 1:1000 | 5 mg/mL, Invitrogen, D21490 | Ex. 405nm (405 diode laser) Em. 410-480nm |
| Anti-GRP78 conjugated to FITC | 1:50 | 200 μg/mL, E-4, Santa Cruz, sc-166490 | Ex. 458nm (Argon laser) Em. 500-535nm |
| Salmonella O antiserum | 1:500 | Rabbit, Difco, 226591 | -- |
| Factor 4 |  |  |  |
| Goat anti-rabbit IgG Rhodamine Red^TM^-X | 1:250 | AB_2338028, Jackson ImmunoResearch, | Ex. 561nm (DPSS 561 laser) |

AffiniPure (secondary)

Rat anti-mouse F4/80 conjugated to PE

Rat anti-mouse F4/80 conjugated to AlexaFluor488

111-295-144

1:100 0.2 mg/mL,

eBioscience, Clone BM8, 12-4801-80

1:150 0.2 mg/mL,

Thermo Fisher, Clone BM8, MF48020

Em. 570-670nm

Ex. 561nm (DPSS 561 laser) Em. 570-670nm

Ex. 488nm (Argon laser) Em. 500-535nm

Congo Red (see methods for curli staining

protocol)

Sigma, C6767 Ex. 633nm

(HeNe 633 laser) Em. 650-755nm

EBBA Biolight 680 1:250 1 mg/mL, EBBA Biotech

Ex. 561nm (DPSS 561 laser) Em. 675-790nm

# SUPPLEMENTARY METHODS

**Curli purification**

Curli aggregates were purified from the *msbB* mutant as previously described [1,2]. Briefly, biofilms were grown by diluting overnight cultures at 1:100 in yeast extract supplemented with casamino acids (YESCA) broth containing 4% DMSO, to enhance biofilm formation and curli production, and grown for 72 hours at 26-28°C with 200 rpm shaking. Aggregates were isolated from the biofilm through treatment with RNAse A from bovine pancreas and DNAse I, sonication, digestion with lysozyme, and boiling to kill bacteria. Then, aggregates underwent a second round of DNAse, RNAse, and lysozyme treatment, followed by boiling again. The biofilm matrix was loaded onto an SDS-PAGE gel that was run overnight; fibrillar aggregates are too large to pass into the gel and can be collected after electrophoresis. The aggregates were washed and sterilized with ethanol, then resuspended in water, and concentration measured by BCA assay according to the manufacturer’s instructions (EMD Millipore, 71285-3). Curli purified in this manner still contains some complexed DNA, despite DNAse treatment steps, due to curli’s ability to protect DNA from digestion [1,3].

# Bone marrow-derived macrophages isolation

BMDMs from C57BL/6 and HLA-B27tg mice were isolated as previously described [4] with some modifications. Briefly, femurs were flushed with RPMI using a sterile syringe and 27-gauge needle, and a single cell suspension of the bone marrow was prepared in RPMI. The suspension was centrifuged at 400 x g for 10 minutes and the pellet was resuspended in BMDM media: 60% RPMI 1640 with 2mM L-glutamine (Gibco, 25030), 1X antibiotic/antimycotic (Gibco, 15240-062), 10% heat-inactivated fetal bovine serum (FBS) (Gibco, 10438-026), and 30% L929 conditioned media which contains M-CSF. The cell suspensions were plated on petri dishes with the BMDM media and grown at 37°C. On day 4, additional BMDM media was added. On day 7, cells were harvested using 0.05% trypsin-EDTA (Gibco, 25300-054) and counted. Cells were then seeded at the appropriate density in the appropriate plates for each assay.

# Preparing coverslips for BMDM cultures

24-well tissue culture plates were prepared: circular coverslips (Fisherbrand, 1254580, 12CIR- 1) were washed briefly with dish soap, tap water, then deionized water to remove dirt from manufacturing. Then, coverslips were acid washed in 1M HCl for 2 hours, rinsed with deionized water, washed three times in 70% ethanol, then flame sterilized and placed into a 24-well plate. Coverslips were coated in sterile 0.1 mg/mL poly-L-lysine at room temperature for 10 minutes. Poly-L-lysine was aspirated and wells were dried in an aseptic cell culture hood for at least 1 hour before BMDMs were seeded at 2x10^6^ cells/well in 500 μl of BMDM media.

# BMDM staining and confocal microscopy

For staining, BMDMs grown on coverslips were washed twice with PBS, fixed for 15 minutes in 4% paraformaldehyde, permeabilized with 0.1% Triton X-100 for 15 minutes, then blocked with 5% BSA for 60 minutes in the dark. Cells were allowed to incubate in anti-GRP78-FITC (**Supplementary Table 1**) overnight at 4°C. The next day, cells were washed twice with PBS, then stained with 5 μg/mL DAPI for 10 minutes. Coverslips were mounted onto Multitest slides (MP Biomedicals, ICN6040805) with VECTASHIELD antifade mounting medium (Vector laboratories, H-1000) and clear nail polish, then imaged using sequential scanning at the appropriate wavelengths (**Supplementary Table 1**) on a TCS SP5 scanning confocal microscope (Leica) using the LAS AF (Leica) software. Images were processed and MFI measured in ImageJ2 (Fiji, Version 2.14.0). Background fluorescence was removed by taking the average MFI for 3 areas without cells per image and subtracting from the cell MFIs from the same image. MFI of curli-treated cells were normalized to the average untreated values from the same batch of BMDMs from the same mouse, due to slight variations in overall brightness of each batch of staining.

# Taconic fecal microbiota transfer

Fecal pellets were collected from female C57BL/6 mice ordered from Taconic farms as donors for microbiota transfer. Feces was collected fresh in sterile PBS, 2 pellets/mL, then homogenized using a wide bore pipet tip and agitation. Fecal homogenate was run through a 100 μm cell strainer to remove large fibers. HLA-B27tg and C57BL/6 mice from Jackson labs were oral gavaged with 200 μl of strained fecal homogenate. Mice were left for 2 weeks to allow colonization of the microbiota before treatment.

# Cecal tissue immunohistochemistry

Slides were sectioned (5 μm) from the same paraffin embedded cecal tissues as for histopathological scoring. Tissue sections were deparaffinized by incubating slides at 60°C for 20 minutes, washing in xylenes (histological, Fisher, X3S-4) twice for 2 minutes, followed by two 1-minute 100% ethanol washes, a 30-second 95% ethanol wash, a 45-second 70% ethanol wash, and a 1- minute deionized water wash to remove excess xylene. A Mini PAP Pen (Genemed, 10-0041) was used to define the area around the tissue on the slide for subsequent steps. Tissue was permeabilized by incubating for 5 minutes in 0.1% Triton-X in PBS, then blocked in 1% BSA in PBS for 30 minutes.

For staining, tissue was incubated for 1 hour in primary antibody cocktail, diluted in blocking buffer (**Supplementary Table 1**), followed by three washes with PBS. Then, when appropriate, tissues spent 1 hour in secondary antibody diluted in blocking buffer (**Supplementary Table 1**), followed by three additional washes with PBS. Tissue was then incubated for 5 minutes with 5 μg/mL DAPI in PBS and washed three times with PBS, before mounting coverslips with VECTASHIELD and clear nail polish. Tissue was imaged as with BMDMs on the Leica confocal microscope using sequential scans at the appropriate spectra for each antibody used (**Supplementary Table 1**).

Images were processed in ImageJ2. Quantification of EBBA+ cells was done on 3 to 4 fields per section per mouse under the 63X objective. Cell counts within a given area were done by first establishing the region of interest (**Supplementary Figure 4A**) and measuring the area. Then all EBBA^+^ cells were selected and EBBA Mean Fluorescence Intensity (MFI) on the red channel and GRP78 MFI on the green channel were quantified in ImageJ2 (**Supplementary Figure 4A**). All MFI were normalized to the average MFI of 5-10 randomly selected EBBA^-^GRP78^Lo^ (cells that appeared to have basal levels of GRP78 staining, not highly excited) from within the lamina propria, excluding cells

that appeared to be epithelial (lined up into neat rows) or red blood cells (no nuclei, characteristic shape, bright green autofluorescence). Next, all selected EBBA^+^ cells were defined as GRP78^Lo^ if the normalized green channel MFI was within 2 standard deviations of the average MFI of EBBA^-^GRP78^Lo^ cells. GRP78^+^ cells were therefore any with a normalized MFI at least 2 std. deviations above negative cells, to eliminate bias based on subjective interpretation of intensity. Correlation of the MFI of EBBA vs. GPR78 can be visualized in **Supplementary Figure 4B**.

Macrophage staining with F4/80-AlexaFluor488 (**Figure 8**) included autofluorescence elimination treatment and antigen retrieval steps. Briefly, after the 30-second 95% ethanol wash during rehydration, transferred slide into 0.25% ammonium chloride dissolved in 70% ethanol for 1 hour, followed by 10 minutes in 70% ethanol, and 1 minute in deionized water, to reduce green autofluorescence of formalin-fixed tissue [5]. Antigen retrieval (Bio-Rad F4/80 Antigen Retrieval protocol) was done by immersing slides in 10 mM sodium citrate buffer, pH 6.0, at 95-100°C for 20 minutes, followed by cooling for 20 minutes in the same buffer, then washing with PBS before continuing with the Mini PAP Pen as above. Blocking buffer was 5% normal horse serum for staining.

F4/80 positivity was quantified in 9 fields each containing EBBA^+^ cells under the 63X objective on one cecal section from each mouse. All EBBA^+^ cells were selected and EBBA Mean Fluorescence Intensity (MFI) on the red channel and F4/80 MFI on the green channel were quantified in ImageJ2 (**Supplementary Figure 6A**). All MFI were normalized to the average MFI of 5-10 randomly selected double negative cells per field. Double negatives were selected from lamina propria cells, avoiding cells that appeared to be epithelium (lined up in neat rows) and red blood cells (no nuclei and very bright green autofluorescence). Then all selected EBBA^+^ cells were defined as F4/80^-^ if the normalized green channel MFI was within 3 std. deviations of the average MFI of double negative cells. F4/80^+^ cells were therefore any with a normalized MFI at least 3 std. deviations above negative cells, to eliminate bias based on subjective interpretation of intensity. Distribution of MFI in positive vs. negative cells can be visualized in **Supplementary Figure 6B**. Total proportion of EBBA^+^ cell counts from HLA-B27tg wild- type STm-infected mice were calculated and graphed in GraphPad Prism (**Figure 8C, Supplementary Figure 6B**).

# SUPPLEMENTARY REFERENCES

1. L.K. Nicastro, S.A. Tursi, L.S. Le, A.L. Miller, A. Efimov, B. Buttaro, V. Tam, Ç. Tükel, Cytotoxic Curli Intermediates Form during Salmonella Biofilm Development, J. Bacteriol. 201 (2019). https://doi.org/10.1128/JB.00095-19.
2. M. Raffatellu, D. Chessa, R.P. Wilson, R. Dusold, S. Rubino, A.J. Bäumler, The Vi capsular antigen of Salmonella enterica serotype Typhi reduces Toll-like receptor-dependent interleukin-8 expression in the intestinal mucosa, Infect. Immun. 73 (2005) 3367–3374. https://doi.org/10.1128/IAI.73.6.3367-3374.2005.
3. S.A. Tursi, E.Y. Lee, N.J. Medeiros, M.H. Lee, L.K. Nicastro, B. Buttaro, S. Gallucci, R.P. Wilson,

G.C.L. Wong, Ç. Tükel, Bacterial amyloid curli acts as a carrier for DNA to elicit an autoimmune response via TLR2 and TLR9, PLoS Pathog. 13 (2017) e1006315. https://doi.org/10.1371/journal.ppat.1006315.

1. C. Tükel, M. Raffatellu, A.D. Humphries, R.P. Wilson, H.L. Andrews-Polymenis, T. Gull, J.F. Figueiredo, M.H. Wong, K.S. Michelsen, M. Akçelik, L.G. Adams, A.J. Bäumler, CsgA is a pathogen-associated molecular pattern of Salmonella enterica serotype Typhimurium that is recognized by Toll-like receptor 2, Mol. Microbiol. 58 (2005) 289–304. https://doi.org/10.1111/j.1365-2958.2005.04825.x.
2. W. Baschong, R. Suetterlin, R.H. Laeng, Control of Autofluorescence of Archival Formaldehyde- fixed, Paraffin-embedded Tissue in Confocal Laser Scanning Microscopy (CLSM), J. Histochem. Cytochem. 49 (2001) 1565–1571. https://doi.org/10.1177/002215540104901210.
